# Fucoxanthin, a Marine Xanthophyll Isolated From *Conticribra weissflogii* ND-8: Preventive Anti-Inflammatory Effect in a Mouse Model of Sepsis

**DOI:** 10.3389/fphar.2019.00906

**Published:** 2019-08-28

**Authors:** Jingqian Su, Kai Guo, Min Huang, Yixuan Liu, Jie Zhang, Lijun Sun, Daliang Li, Ka-Lai Pang, Guangce Wang, Long Chen, Zhiyu Liu, Youqiang Chen, Qi Chen, Luqiang Huang

**Affiliations:** ^1^Fujian Key Laboratory of Innate Immune Biology, Biomedical Research Center of South China, College of Life Science, Fujian Normal University, Fuzhou, China; ^2^The Public Service Platform for Industrialization Development Technology of Marine Biological Medicine and Product of State Oceanic Administration, Center of Engineering Technology Research for Microalgae Germplasm Improvement of Fujian, Southern Institute of Oceanography, Fujian Normal University, Fuzhou, China; ^3^Department of Molecular Biology, University of Texas Southwestern Medical Center, Dallas, TX, United States; ^4^Institute of Marine Biology and Center of Excellence for the Oceans, National Taiwan Ocean University, Keelung, Taiwan; ^5^Institute of Oceanology, Center for Ocean Mega-Science, Chinese Academy of Sciences, Qingdao, China; ^6^Laboratory for Marine Biology and Biotechnology, Qingdao National Laboratory for Marine Science and Technology, Qingdao, China; ^7^Division of Neurocritical Care, Huashan Hospital, Fudan University, Shanghai, China; ^8^Fisheries Research Institute of Fujian, Fujian Collaborative Innovation Center for Exploitation and Utilization of Marine Biological Resources, Key Laboratory of Cultivation and High value Utilization of Marine Organisms in Fujian Province, Xiamen, China

**Keywords:** fucoxanthin,lipopolysaccharide, nuclear factor-κB, sepsis, Conticribra weissflogii

## Abstract

**Background:** Fucoxanthin (FX), a xanthophyll pigment which occurs in marine brown algae with remarkable biological properties, has been proven to be safe for consumption by animals. Although FX has various pharmacological effects including anti-inflammatory, anti-tumor, anti-obesity, antioxidant, anti-diabetic, anti-malarial, and anti-lipid, *in vivo* protective effect against sepsis has not been reported. In this study, we aimed at evaluation the efficacy of the FX in a model of sepsis mouse.

**Methods:** FX was successfully isolated from *Conticribra weissflogii* ND-8 for the first time. The FX was identified by thin-layer chromatography (TLC), high-performance liquid chromatography-mass spectrometry (HPLC-MS), and nuclear magnetic resonance (NMR). Animals were randomly divided into 9 groups, including Sham group (mouse received an intraperitoneal injection of normal saline 1.0 ml/kg), FX-treated (0.1–1.0 ml/kg), Lipopolysaccharide (LPS)-treated (20 mg/kg), FX+LPS-treated (0.1–10.0 mg/kg and 20 mg/kg, respectively), and urinastatin groups (10^4^ U/kg). Nuclear factor (NF)-κB activation could be potential treatment for sepsis. NF-κB signaling components were determined by western-blotting. IL-6, IL-1β, TNF-α production, and NF-κB activation were evaluated by ELISA and immunofluorescent staining *in vitro*.

**Results:** FX was found to decrease the expression of inflammatory cytokines including IL-6, IL-1β, and TNF-α, in a prophylactic manner in the LPS-induced sepsis mouse model. Meanwhile, FX significantly inhibits phosphorylation of the NF-κB signaling pathway induced by LPS at the cellular level and reduces the nuclear translocation of NF-κB. The IC_50_ for suppressing the expression of NF-κB was 11.08 ± 0.78 μM in the THP1-Lucia™ NF-κB cells. Furthermore, FX also inhibits the expression of inflammatory factors in a dose-dependent manner with the IC_50_ inhibition of IL-6 production was 2.19 ± 0.70 μM in Raw267.4 macrophage cells. It is likely that the molecules with the ability of targeting NF-κB activation and inflammasome assembly, such as fucoxanthin, are interesting subjects to be used for treating sepsis.

## Introduction

Fucoxanthin (FX) is an important carotenoid present in algae, such as edible brown algae and diatoms, and was first isolated from brown seaweeds. Currently, the price of FX standard products is about 30,000 dollars per gram. In recent years, the origin, extraction, and purification methods, biosynthetic pathways, physiological activities, and metabolic modes of FX have been extensively studied. FX has a unique structure and exhibits potential advantages with a variety of pharmacological activities, including anti-inflammatory, anti-tumor, anti-obesity, antioxidant, anti-diabetic, anti-malarial, and anti-lipid effects (Maeda, 2015). Thus, FX possesses a potential value for further pharmaceutical development.

FX is widely distributed in algae and some invertebrate cells, including members of the Heterokontophyta and Haptophyta families. Among them, diatoms and algae, especially brown algae, are rich in FX. FX in algae accounts for more than 10% of the total carotenoid produced in nature (Xia et al., 2013). Currently, the raw materials used for FX extraction are often derived from brown algae, including kelp, sargassum, and wakame, which grow seasonally. There is 16.51 mg/gdw FX content in *Phaeodactylum tricornutum* under photo-culture model with aeration agitation (Kim et al., 2012a), over 18 mg/gdw FX in *Isochrysis galbana* under photo-culture model with aeration (Kim et al., 2012b), and 0.033 mg/gdw FX in *Laminaria japonica* (Xiao et al., 2012). However, FX is only present in the surface cortical cells of the brown algae at a low concentration and its production efficiency is very low. In addition, chemical synthesis of FX is very difficult. Therefore, efficiently producing FX and understanding its pharmacological functions play an essential role for further exploring its economic value and facilitating its widespread use.

*Conticribra weissflogii* is a fast growing, single-celled diatom that can be cultivated during all four seasons and artificially cultured in a photoreactor. However, FX has not been shown to previously be produced from *C. weissflogii*.

Lipopolysaccharide (LPS) is the prototypical endotoxin and is present in the outer membrane of Gram-negative bacteria (Palsson-McDermott and O’Neill, 2004). LPS can tcause strong immune responses by promoting the secretion of pro-inflammatory cytokines on immune cells (Iwasaki and Medzhitov, 2015), it has been commonly used in animal models of sepsis (Remick et al., 2000). During sepsis, the homeostasis between pro-inflammatory and anti-inflammatory cytokines is disrupted, resulting in the release of inflammatory factors, such as IL-6, IL-1β, and TNF-α (Weber et al., 2015). Thus, reducing the inflammatory response might be a feasible strategy to deal with the systemic inflammatory response syndrome for treating sepsis as suggested previously (Delano and Ward, 2016). Interestingly, it was found that FX can significantly inhibit ear swelling and decrease TNF-α level, suggesting that FX induces anti-inflammatory effects by inhibiting the degranulation of mast cells *in vivo* (Sakai et al., 2011). The results revealed that FX inhibited LPS-induced uveitis by inhibiting inducible NO expression of enzymes and cyclooxygenase-2 protein (Shiratori et al., 2005). However, whether FX is an effective modulator in sepsis have not yet been reported, and the mechanisms associated with this function is unknown.

In the present study, we developed a procedure to extract purified FX from cultured *C. weissflogii* ND-8. We further evaluated the effects of FX on LPS-mediated inflammatory response in a cell model, investigated the protective effect of FX in LPS-induced sepsis mouse model, and explored the signal transduction mechanism related to its anti-inflammatory effects.

## Materials and Methods

### Chemicals and Reagents

All chemicals were obtained from from Sigma (St. Louis, MO, USA), unless otherwise stated.

### Diatom Materials and Sample Preparation

*C. weissflogii* ND-8 was isolated from the coastal water of Zhoushan, Zhejian Province in China. It was cultured in Guillard’s f/2 medium prepared from filtered, sterilized natural seawater, with an inoculation ratio of 1:8, under 12 h light condition (light intensity of 75 μmol/m^2^/s) and 12 h dark time in 1 day at 20°C–22°C.

### Characterization of *C. weissflogii* ND-8

The photomicrographs of *C. weissflogii* ND-8 were taken with an optical microscope (FM 10 Camera; Nikon, Tokyo, Japan) and a scanning electron microscope (JSM-6380LV, JEOL, Tokyo, Japan). Molecular identification was performed as previously described (Su and Yang, 2015). Primers ITS5-F (5’-TCACCTACGGAAACCTTGT-3’)/ITS5-R (5’-TTCAGCGGGTAGTCTTGCCTC-3’), and 18S-F (5’-ACCTGGTTGATCCTGCCAGT-3’)/18S-R (5’-TCACCTACGGAAACCTTGT-3’) were used to amplify the ND-8 ITS and 18S fragments, respectively. Their ITS and 18S sequences were compared with those available in the NCBI databases using BLAST. The search results were processed with the MEGA5.2 software (Tamura et al., 2011). The phylogenetic tree was constructed by the neighbor-joining method, 1,000 replications of random search were carried out to assess the reliable level of the tree (Saitou and Nei, 1987).

### FX Extraction and Isolation

*C. weissflogii* ND-8 was grown in Guillard’s f/2 medium at 20°C–22°C for 5 days, followed by centrifugation at 4,000 × *g* for 15 min. The algae mud was collected, freeze-dried at –70°C for 2 days. The purification of FX was performed as previously described (Xia et al., 2013). Additionally, to avoid interference of light, all experiments were performed in the dark. The active fractions were pooled by TLC in a solvent system containing petroleum ether/ethyl acetate, 1:1 (v/v). The retention factor (R_f_) was calculated as follows:

$${\text{R}}_{\text{f}}=\frac{\text{distance traveled by the compound}}{\text{distance traveled by the solvent front}}$$

The active fractions were pooled and concentrated in vacuum. The concentrates were finally dried with a nitrogen blower for subsequent separation and analysis.

### HPLC-MS

HPLC-MS analysis was performed on the *Thermo* HPLC-MS system (Thermo Scientific, Waltham, MA, USA) using the Thermo Hypersil GOLD C_18_ column (1.9-μm particle size, 2.1 mm × 100 mm) with methanol and water as eluents. The experimental conditions were following: injection volume: 5 μM; mobile phase: 0–0.2 min, 95% B; 0.2–3.5 min, 95%–2% B; 3.5–5 min, 2% B; 5–7.5 min, 2%–95% B; 7.5–10 min, 95% B; flow rate: 0.3 ml·min^–1^. The HPLC eluate was administered to the MS system with a spray voltage of 1.0 kV. The MS peaks were recorded and compared with that of the FX standard.

### NMR

The isolated target sample (2.0 mg) and standard FX (2.0 mg) were dissolved in 0.5 ml of deuterochloroform (CDCl_3_) and the ^1^H nuclear magnetic resonance (NMR) was measured using the Bruker 400 MHz NMR spectrometer (MA, USA).

### Animals and Treatments

Specific pathogen-free C57BL/6 adult mice aged 8–10 weeks old and weighing 20 ± 1 g were purchased from the Fujian Medical University Animal Facility (Fujian, China). The sample included 126 animals, half male and half female. All animal experiments were conducted in accordance to the Guide for the Care and Use of Laboratory Animals approved by the Fujian Provincial Office for Managing Laboratory Animals and was guided by the Fujian Normal University Animal Care and Use Committee (Approval No. 201800013).

After acclimation for 1 week, 90 mice were randomly divided into nine groups (*n* = 10/group) with half male and half female, including Sham group (mouse received an intraperitoneal injection of normal saline 1.0 ml/kg), FX-treated (0.1–10.0 mg/kg), LPS-treated (20 mg/kg), FX+LPS-treated (0.1–10.0 mg/kg and 20 mg/kg, respectively), and urinastatin groups (10^4^ U/kg). LPS was obtained from *Escherichia coli* 0111:B4 cells (Cell Signaling Technology, Beverley, MA, USA). Ulinastatin was used as the positive control. The sham group was injected with the same amount of phosphate-buffered saline (PBS) (0.0067M; pH 7.4, HyClone, GE Healthcare Life Sciences, UT, USA). The mice were intraperitoneally (ip) injected with FX 30 min before the ip administration of a lethal dose of LPS (20 mg/kg) or PBS. All mice were fasted for 12 h preoperatively, but were free to drink water. The survival rate of mice was recorded every 6 h for 120 h, and Kaplan–Meier survival curves were generated using the GraphPad Prism 6 software (v.5.01 for Windows; GraphPad Software, San Diego, CA, USA) and analyzed by the log-rank test. Based on the above experiments, the other 36 mice were randomly divided into six groups (*n* = 6/group) with half male and half female. After anesthetization using Pentobarbital sodium salt, mouse blood was drawn *via* retro-orbital puncture 6-h post-challenge and allowed to clot at 28°C for 30 min. The serum was subsequently collected by centrifugation at 2000 ×*g* for 30 min and stored at –80°C for further analysis. The mice tissues were collected for further analyses.

### Histopathological Examination

Mouse tissues were fixed with paraformaldehyde (4%) in PBS for histological analysis. Tissues were rinsed with water, dehydrated with ethanol, and embedded in paraffin, followed by cryostat sectioning (∼4 μm) and mounting onto glass slides. Sections were then dewaxed using xylene and ethanol, and stained with general hematoxylin and eosin (H&E) to reveal hemorrhagic necrosis in the tissues. Histological changes were observed under a light microscope (Olympus, Japan) at 100× and 200× magnifications. According to Eriksson et al., hepatic injury score was measured on the H&E-stained sections using grades from 0 to 4 as follows: a score of 0 represented no inflammatory infiltrates; 1 represented small inflammatory cells between hepatocytes; 2 represented larger foci of >100 inflammatory cells; 3 represented >10% of a cross section involved; 4 represented >30% of a cross section involved (Eriksson et al., 2003).

### Cell Culture

Murine macrophage RAW 264.7 was purchased from the American Type Culture Collection (Manassas, VA, USA) and used as an in vitro model to investigate the anti-inflammatory properties of FX. The cells were cultured in Dulbecco’s modified Eagle medium containing 10% (v/v) fetal bovine serum (Gibco, CA, USA) and 1% (v/v) penicillin/streptomycin (Gibco, CA, USA) at 37°C in a humidified incubator with 5% carbon dioxide (CO2).

### Cell-Viability Assay

Cell viability was evaluated in RAW 264.7 cells using the Cell Counting Kit-8 (CCK-8; Beyotime Biotechnology, Beijing, China). The absorbance of the sample at 450 nm was measured using a microplate reader (Synergy HT; BioTek, Winooski, VT, USA), and the percent of surviving cells in each treated group was plotted.

### Real-Time Quantitative PCR

Cytokine mRNA expression and secretion was measured by the real-time quantitative polymerase chain reaction following reverse transcription (RT-qPCR). The total RNA was separated using TRIZOL (Invitrogen, Carlsbad, CA, USA). The RT-qPCR was performed using SYBR Green (Applied Biosystems, Foster City, CA, USA) as previously described (Fan et al., 2018). The primers used for RT-qPCR were following: IL-1β-F (5’-ACAGGCTCCGAGATGAACAA-3’)/IL-1β-R (5’-TGGGAGTAGACAAGGTACAACCC-3’), IL-6-F(5’-TAGTCCTTCCTACCCCAATTTCC-3’)/IL-6-R (5’-CCTCTCGGCAGTGGATAAAG-3’), and TNF-α-F (5’-CATCTTCTCAAAATTCGAGTGACAA-3’)/TNF-α-R (5’-TGGGAGTAGACAAGGTACAACCC-3’).

### Measurement of Pro-Inflammatory Cytokine Levels by ELISA

RAW 264.7 cells were pre-treated with FX at the indicated doses for 6 h followed by 3-h LPS (1.0 mg/L) treatment, and grown in 24-well plates (1 × 10^6^ cells/well) for 24 h. The supernatants of the cultured RAW 264.7 cells were collected.

The levels of TNF-α, IL-1β, and IL-6 in the supernatants and serum samples (see above) were quantified using the ELISA kits (TNF-α, R&D Systems, Minneapolis, MN, USA, catalog number SMTA00B; IL-1β, R&D Systems, Minneapolis, MN, USA, catalog number SMLB00C; IL-6, R&D Systems, Minneapolis, MN, USA, catalog number SM6000B) according to manufacturer’s protocols.

### Western Blot Analysis

Western blotting was performed as previously described (Chen et al., 2002; Xiao et al., 2019). Antibodies anti-myeloid differentiation primary response gene 88 (MyD88) (D80F5; #4283), anti-phospho-IKKα/β (Ser176/180) (16A6; #2697), anti-IKKα (#2682), Phospho-IKKα/β (Ser176/180) (16A6, #2697), anti-IκBα (44D4,#4812), anti-Phospho-IκBα (Ser32/36) (5A5, #9246), anti-NF-κB p65 (D14E12, #8242) and anti-Phospho-NF-κB p65 (Ser536) (93H1, #3033) were purchased from Cell Signaling Technology.

### NF-κB Luciferase Activity Assay

Human THP1-Lucia^TM^ NF-κB cells were derived from the human THP-1 monocyte cell line by stable integration of an NF-κB-inducible Lucia^TM^ reporter construct. THP-1 Lucia NFκB reporter cells were purchased from InvivoGen (San Diego, CA, USA). THP1-Lucia^TM^ NF-κB cells were specifically designed for monitoring the NF-κB signal transduction pathway in a physiologically relevant cell line. The THP1-Lucia^TM^ NF-κB cells were grown on 96-well plate (1 × 10^5^/well) 18 h in the presence of the different concentrations FX followed by LPS (1.0 mg/L) for stimulation. For the determining of the luciferase activity, the 20 μl aliquots of cell culture media were relocated into the 96-well black plates (Corning, NY, USA) followed by QUANTI-Luc^TM^ assay solution (InvivoGen). Plates were measured immediately for luciferase activity with Victor 2 multiplate reader (PerkinElmer) according to the manufacturer’s instructions.

### Immunofluorescent Staining

Cells were grown and fixed with 4% paraformaldehyde for 10 min at room temperature, followed by treatment with membrane penetration solution (0.3% Triton-100) for 10 min at room temperature. The cells were washed with 1 × PBS five times then incubated with anti-NF-κB (p65) primary antibodies (1:200 dilution) (Cell Signaling Technology, USA) overnight at 4°C, followed by incubation with AlexaFluor 488 goat antirabbit secondary antibody at 37°C in the dark for 30 min. Nuclei were counterstained with 0.5 μg/ml 4′,6-diamidino-2-phenylindole (DAPI) (1:800, Santa Cruz) in PBS for 2 min. Negative controls were prepared by omitting primary antibodies. After washing with PBS three times, samples were mounted in mounting medium (M1289, Sigma-Aldrich), observed under a Zeiss fluorescence microscope (Carl Zeiss, Oberkochen, Germany), and image analyses were performed using Zeiss LSM 510 software.

### Statistical Analyses

The data are expressed as mean ± standard deviation (SD). Statistical significance was determined by the one-way ANOVA and Tukey’s test for *post hoc* multiple comparison by 5 software. The *P* value < 0.05 was considered statistically significant.

## Results

### Morphological and Molecular Identification of Strain *C. weissflogii* ND-8

**Figure 1:**
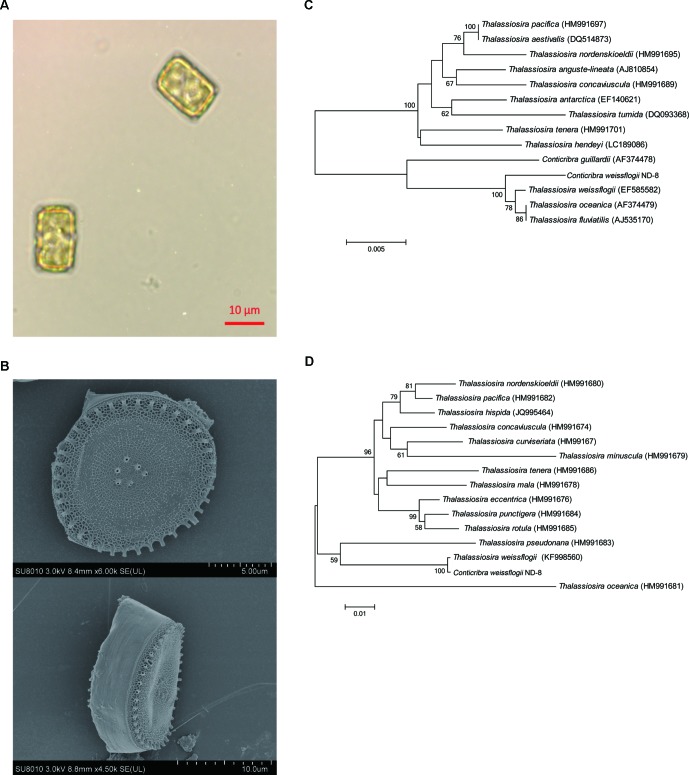
Morphological and molecular identification of *Conticribra weissflogii* ND-8. **(A)** Morphology of *C. weissflogii* ND-8 and **(B)** intact cells observed using a scanning electron microscope. Scale bars: **(A)** 10 μm; **(B)** 2 μm. **(C)** The neighbor-joining tree of the 18S rDNA sequences of *C. weissflogii* ND-8 and **(D)** the neighbor-joining tree of the internal transcribed spacer (ITS) rDNA sequences of *C. weissflogii* ND-8. The numbers at the nodes are the bootstrap scores (above 90%) obtained from 1,000 replications.

The morphology of *C*. *weissflogii* ND-8 was observed under microscope. Valves are circular with flat valve face, and valve diameter is 10–18 μm (**Figure 1A**). The scanning electron microscopy photos showed that marginal fultoportula located on mantle and 5–7 fultoportula are present near the valve center (**Figure 1B**). The morphological features of ND-8 were consistent with those of *Conticribra* sp.

To further verify the identification of ND-8 strain, its 18S and ITS rDNA sequences (GenBank accession no. GQ414523) were analyzed using the blast search in NCBI. The molecular data in the present paper suggested that *C. weissflogii* (ND-8) and other Conticribra species form a monophyletic group separated from some species of Thalassiosira, which was described as a new species from three additional areas of Korea (Park and Lee, 2014). In 18S analysis, *C. weissflogii* (ND-8) clustered with *C. weissflogii* (EF585582) (**Figure 1C**), *T. oceanica* (AF374479) and *T. fluviatilis* (AJ535170), and with a sister clade comprising *C. guillardii* (AF374478). In ITS analysis (**Figure 1D**), *C. weissflogii* (ND-8) clustered with *C. weissflogii* (KF998560). Thus, the strain ND-8 was assigned to the species *C. weissflogii*.

### Isolation and Identification of FX in ND-8

**Figure 2:**
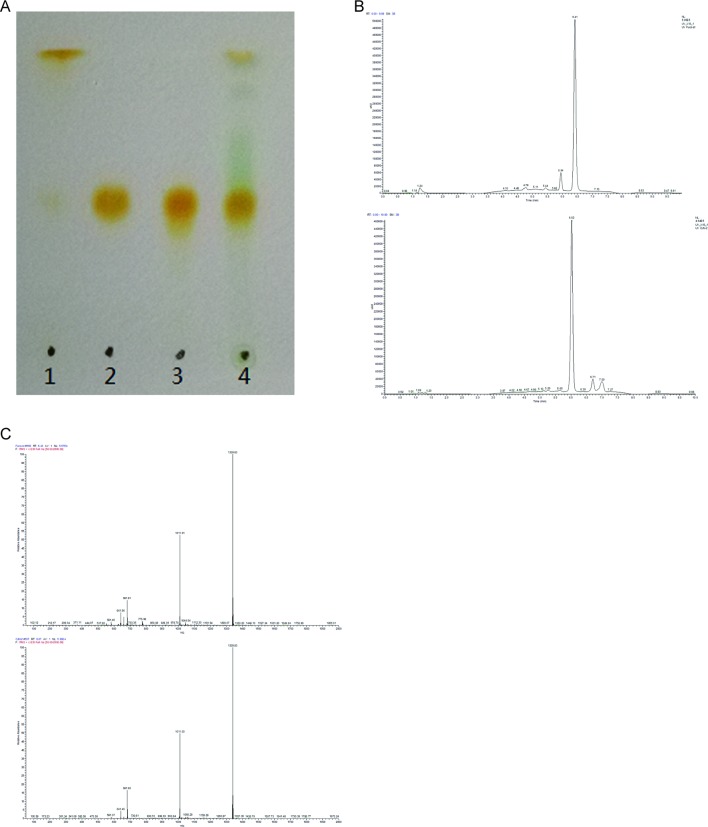
**(A)** Thin-layer chromatography of standard β-carotene (lane 1), standard fucoxanthin (FX) (lane 2), the purified FX sample of ND-8 (lane 3), and the methanol and acetone extract of ND-8 (lane 4) on a silica plate. **(B–C)** High-performance liquid chromatogram and mass spectra of standard FX and ND-8 FX eluted by the silica gel column chromatography; the mobile phases used were methanol and deionized water, the mobile phase flow rate was 0.3 ml.min^–1^. Retention times: standard FX, 6.41 min; ND-8 FX, 6.02 min. The diagnostic ions of standard FX are at *m*/*z* 641.46 (M + H-HOH)^+^, 681.81 (M + Na)^+^, and 1,339.83 (2M + Na)^+^. The diagnostic ions of ND-8 FX are at *m*/*z* 641.46 (M + H-HOH)^+^, 681.80 (M + Na)^+^, and 1,339.83 (2M + Na)^+^.

**Figure 3:**
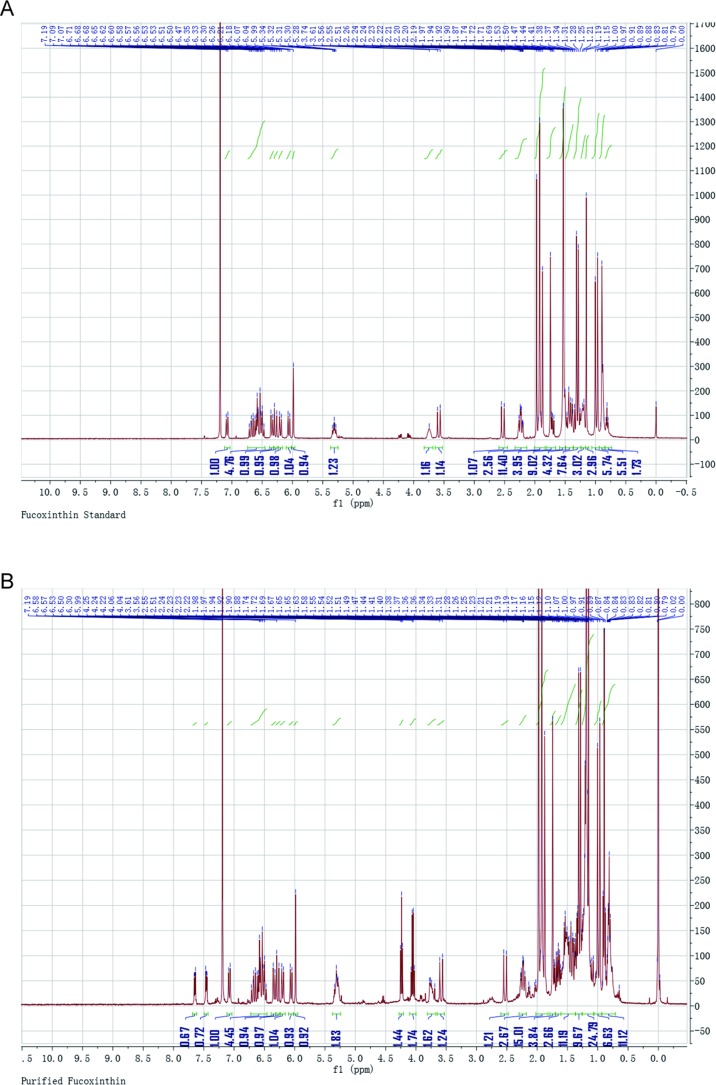
Nuclear magnetic resonance spectroscopy of the standard fucoxanthin (FX) **(A)** and ND-8 FX **(B)**.

The presence of FX in the supernatant of ND-8 culture was examined by chromatographic and spectroscopic analyses. The thin-layer chromatographic pattern of separated components was similar to that of FX (R_f_, 0.396) (**Figure 2A**). The spectrometric profiles including the peak position and peak shape of the compound derived from ND-8 were similar to those of the standard FX, with a retention time of 6.41 min (**Figure 2B**). Furthermore, the identity of the putative FX was verified by electrospray mass spectroscopy. The standard FX produces a characteristic (M + H-HOH)^+^ peak at 641.46, (M + Na)^+^ peak at 681.81, and (2M + Na)^+^ peak at 1339.83 (Rajauria et al., 2017). The purified compound from ND-8 produced an identical electrospray mass spectrum, with the following major molecular ions: (M + H-HOH)^+^ peak at 641.46, (M + Na)^+^ peak at 681.80, and (2M + Na)^+^ peak at 1339.83 (**Figure 2C**). The results of NMR analysis of the standard FX and purified compound from ND-8 are as follows: 1) As shown in **Figure 3A**, the NMR analysis data of standard FX: ^1^H NMR (400 MHz, CDCl_3_) 7.07 (d, *J* = 8 Hz, 1H), 6.47–6.71 (m, 4–5 H), 6.33 (d, *J* = 8 Hz, 1H), 6.26 (d, *J* = 16 Hz, 1H), 6.18 (d, *J* = 12 Hz, 1H), 6.04 (d, *J* = 12 Hz, 1H), 5.99 (s, 1 H), 5.28–5.34 (m, 1 H), 3.74 (s, br, 1 H), 3.56 (d, *J* = 20 Hz, 1H), 2.51 (d, *J* = 16 Hz, 1H), 2.19–2.28 (m, 2 H), 1.87–1.97 (m, 9–11 H), 1.69–1.74 (m, 4 H), 1.36–1.54 (m, 4–5 H), 1.17–1.34 (m, 10 H), 1.15 (s, 3 H), 0.97 (d,*J* = 12 Hz, 5 H), 0.88 (d, *J* = 4 Hz, 5 H). MS (ESI) calculated for [C_42_H_59_O_6_]^+^ ([M + H]^+^): 659.4, found: 659.3 [M + H]^+^, 682.0 [M + Na]^+^, 1,340.0 [2M + Na]^+^. (2) As shown in **Figure 3B**, the NMR analysis data of the purified compound from ND-8: ^1^H NMR (400 MHz, CDCl_3_) 7.64–7.66 (m, 1 H), 7.45–7.47 (m, 1 H), 7.07 (d, *J* = 8 Hz, 1H), 6.47–6.71 (m, 4–5 H), 6.33 (d, *J* = 8 Hz, 1H), 6.26 (d, *J* = 16 Hz, 1H), 6.18 (d, *J* = 12 Hz, 1H), 6.04 (d, *J* = 12 Hz, 1H), 5.99 (s, 1 H), 5.28–5.34 (m, 2 H), 4.24 (t, *J* = 6 Hz, ∼ 2 H), 4.03 (q, *J* = 8 Hz, ∼ 2 H, Ethyl Acetate), 3.74–3.77 (m, br, 1–2 H), 3.56 (d, *J* = 20 Hz, 1H), 2.51 (d, *J* = 16 Hz, 1H), 2.19–2.28 (m, 2 H), 1.87–1.97 (m, 9–11 H), 1.69–1.74 (m, 4 H), 1.36–1.54 (m, 4–5 H), 1.17–1.34 (m, 10 H), 1.15 (s, 3 H), 0.97 (d,*J* = 12 Hz, 5 H), 0.88 (d, *J* = 4 Hz, 5 H), 0–2 (solvent residue). MS (ESI) calculated for [C_42_H_59_O_6_]^+^ ([M + H]^+^): 659.4, found: 659.3 [M + H]^+^, 681.8 [M + Na]^+^, 1339.5 [2M + Na]^+^.

There is a difference in retention time between the standard FX (6.41 min) and the homemade FX (6.02 min), which might be caused by the different separation protocol. Their NMR data and MS spectra showed identical. In this study, the purity of the FX isolated from *C. weissflogii* is 91% and there is 6 mg/gdw FX under photo-culture model without aeration agitation. Based on these data, we conclude that *C. weissflogii* ND-8 synthesizes FX *in vivo*.

### FX Treatment Improves the Survival Rate of LPS-Treated Mice

**Figure 4:**
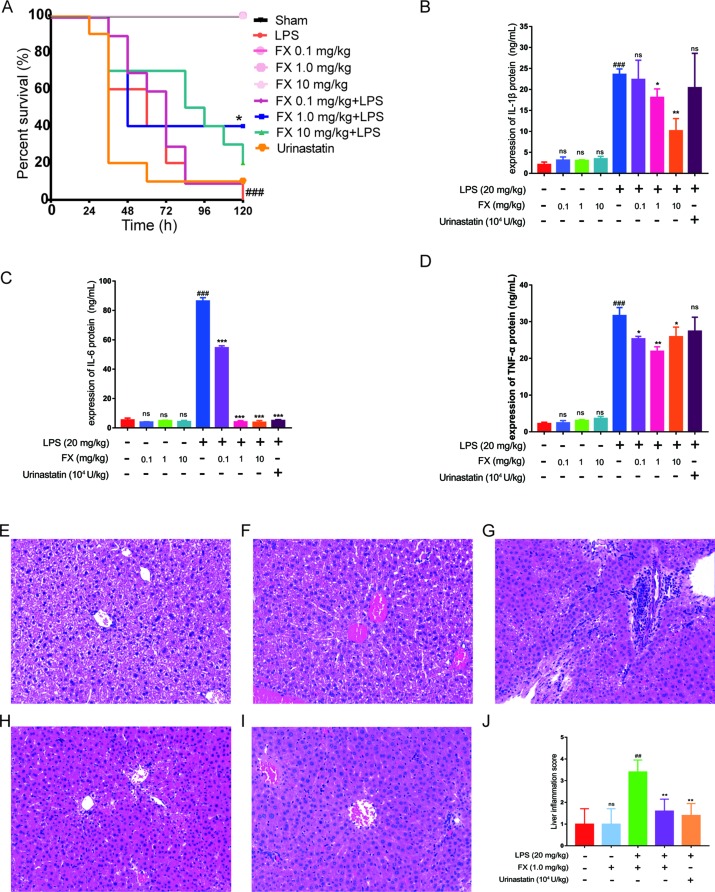
Effects of FX on LPS-induced sepsis in mice. The mice were treated with FX (0.1–10 mg/kg) 2 h before the administration of lipopolysaccharide (LPS) (20 mg/kg, ip). Ulinastatin (10^4^ U/kg) was used as a control. **(A)** The effects of different doses of FX on the survival of LPS-treated mice (n = 10). **(B, C, D)** The serum levels of the inflammatory factors TNF-α, IL-1β, and IL-6 were measured using the ELISA kits (n = 6). The effect of FX on liver tissue histopathological changes in LPS-induced septic mice. Histopathologic sections of liver tissues (H&E, ×200). **(E)** Liver tissues from control, **(F)** FX (1.0 mg/kg), **(G)** LPS (20 mg/kg), **(H)** LPS (20 mg/kg) + FX (1.0 mg/kg), **(I)** LPS (20 mg/kg) + Urinastatin groups (10^4^ U/kg) and **(J)** the pathological scores was evaluate in LPS-induced septic mice. The data were analyzed by the one-way ANOVA, followed by Tukey’s *post hoc* test. (##) *p* < 0.01, (###) *p* < 0.001, significantly different when compared with that of the control group, ns: not significant. (∗) *p* < 0.05, (∗∗) *p* < 0.01, and (∗∗∗) *p* < 0.001, significantly different when compared with that of the LPS group.

Next, we evaluated the functional role of the above isolated FX in a LPS-treated sepsis mouse model. Thirty minutes post challenge with LPS, the mice developed symptoms including shortness of breath, refusal of food intake, and increased eye secretion. These conditions worsened over time, with death occurring after 12 h. As shown in **Figure 4A**, the overall survival rate with the high-dose FX-treatment (10.0 mg/kg) was 20% in the LPS-treated mice. In contrast, the survival rate of mice in the LPS plus 1.0 mg/kg FX-treated group was higher than that observed in the LPS-treated group (20 mg/kg) (*p < 0.05*), exhibiting an increase in the survival rate from 0% to 40%. Additionally, the survival rate of mice in the three groups treated with FX alone was 100%, indicating that FX treatment resulted in no apparent toxicity in mice. Ulinastatin which has been clinically used for relieving inflammation in many disease including was also used as a control. However, we did not observe the improvement of mouse survival treated with ulinastatin. At the same time, we did not found any gender effect. These results suggest that FX (1.0 mg/kg) protects the mice against LPS-induced sepsis death. From these results, we determine that 1.0 mg/kg was the appropriate dosage for FX treatment and was selected for further analyses.

### FX Treatment Suppressed Inflammatory Cytokine Production in LPS-Treated Mice

A major hallmark of sepsis is the production of large amount of inflammatory cytokines (Bosmann and Ward, 2013; Kanashiro et al., 2017). As LPS-induced death is highly correlated with inflammation, we examined the inflammatory cytokine levels in the sera of LPS-treated mice. All tests were performed 6 h after the administration of LPS with or without FX treatment (Zahar et al., 2011), with ulinastatin (10^4^ U/kg) as a control. The serum levels of inflammatory factors including TNF-α, IL-1β, and IL-6 were measured using the ELISA kits. As shown in **Figures 4B, C, and D**), FX (0.1, 1.0 and 10 mg/kg) alone treatments did not affect the serum levels of these cytokines, whereas the serum levels of TNF-α, IL-1β, and IL-6 of the LPS group were all significantly increased (*p < 0.01*). In contrast, the FX treatment (1.0 mg/kg, ip) significantly reduced the serum levels of TNF-α, IL-1β, and IL-6 induced by LPS (*p < 0.05*). Urinastatin treatments did not affects the serum levels of these cytokines except the IL-6. These results suggest that FX exhibits anti-inflammatory effects in LPS-induced septic mice.

Septic mice were sacrificed at 6 h post-administration of LPS, and histopathology changes of liver tissues from each experimental group were determined by H&E staining. In the control and FX (1.0 mg/kg) groups, the liver sections showed normal cell morphology with no infiltration of inflammatory cells observed (**Figures 4E, F**). However, the liver tissues from LPS (20 mg/kg)-treated group showed clear pathological changes with swelling cells and infiltration of inflammatory cells (**Figure 4G**), whereas symptoms of those histopathological damage were significantly alleviated in the LPS (20 mg/kg) + FX (1.0 mg/kg) groups and LPS (20 mg/kg) + Urinastatin (10^4^ U/kg) group (**Figures 4H, I**). In addition, the scores were evaluated to determine the degree of histopathological damage. As it is shown in **Figure 4J**, the mean pathological score was significantly increased from 1.0 ± 0.4 to 3.4 ± 0.24 after LPS (20 mg/kg) administration compared with control group. However, the pathological scores were reduced by treatments of FX (1.0 mg/kg) and Urinastatin (10^4^ U/kg) (1.6 ± 0.24 and 1.4 ± 0.24).

### FX Inhibits Inflammatory Cytokine Expression in LPS-Treated RAW264.7 Cells

**Figure 5:**
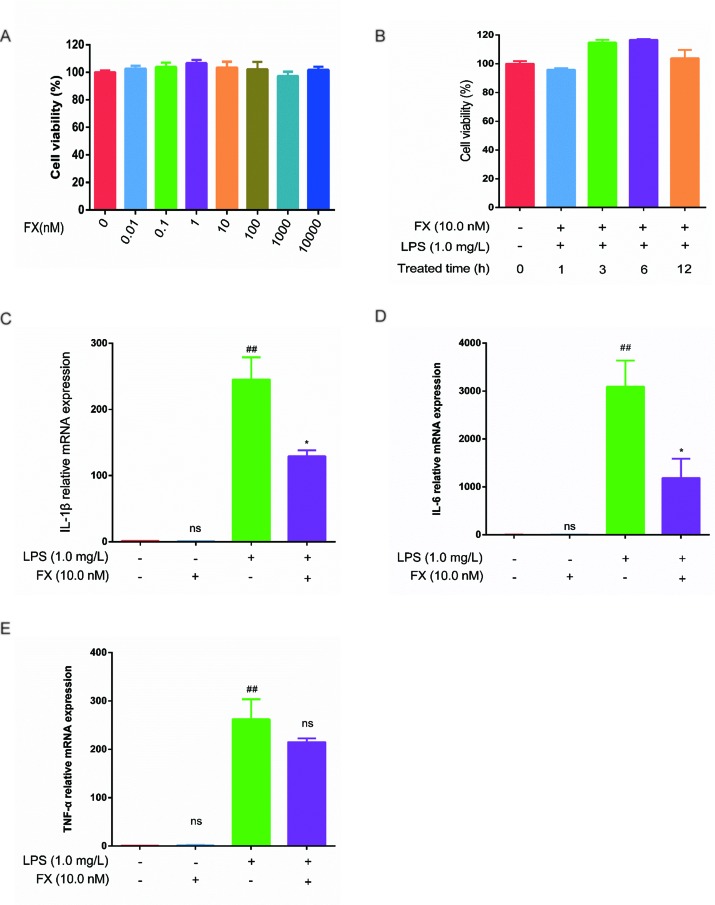
Effects of FX on LPS-treated RAW264.7 cells. **(A)** Effect of FX treatment (0–10 μM) on cell viability. **(B)** Effect of FX (10.0 nM) + LPS (1.0 mg/L) co-treatment on cell viability. **(C**, **D**, and **E)** The qPCR was performed to test the changes in cytokine mRNA levels (IL-1β, IL-6, and TNF-α) in RAW264.7 cells following FX treatment alone or co-treatment with LPS. The data were analyzed by the one-way ANOVA, followed by Tukey’s *post hoc* test. (##) *p* < 0.01, significantly different when compared with that of the control group, ns: not significant. (∗) *p* < 0.05, significantly different when compared with that of the LPS group.The data are representative of three independent experiments.

To verify the FX effects on the cellular level, we first tested the FX effect on cell viability. As shown in **Figure 5A**, FX (0–10 μM) treatment had no apparent effect on cell viability. Previously, FX at the concentration of 10.0 nM has been shown to exhibit functional effects at the cellular level, and it did not show any toxicity (Peng et al., 2011). In line with this, as shown in **Figure 5B**, the FX (10.0 nM) treatments at different intervals (0–12 h) also showed no effect on cell viability (*p >* 0.05), therefore, we chose 10.0 nM to perform the subsequent analyses.

Next, we examined the effects of FX on cytokine production induced by LPS treatments in RAW264.7 cells. RAW264.7 cells were incubated with FX (10.0 nM) for 6 h, followed by the 3-h LPS (1.0 mg/L) treatment. Changes in the expression of TNF-α, IL-1β, and IL-6 were tested by RT-qPCR. As shown in **Figure 5** (**C**, **E**), the mRNA levels of TNF-α, IL-1β, and IL-6 were significantly elevated (*p <* 0.01) by LPS. In contrast, the mRNA levels of TNF-α, IL-1β, and IL-6 induced by LPS were significantly suppressed in the FX+LPS group (*p* < 0.01). No change in the mRNA levels of TNF-α, IL-1β, and IL-6 was observed in the cells treated with FX alone. These results suggest that the FX (10.0 nM) treatment significantly inhibited LPS-induced upregulation of TNF-α, IL-1β, and IL-6 transcription in RAW264.7 cells.

**Figure 6:**
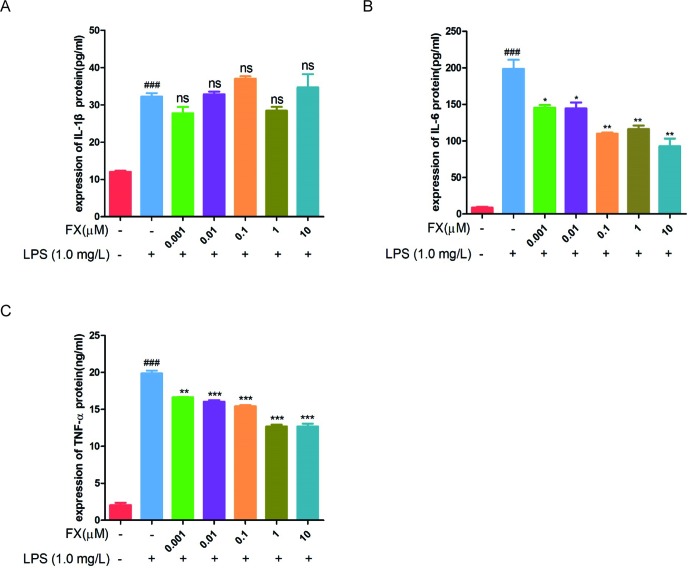
ELISA assays were performed to test the changes in cytokine protein levels in RAW264.7 cells following FX treatment at the indicated doses or co-treatment with LPS (1.0 mg/L). **(A)** IL-1β, **(B)** IL-6, and **(C)** TNF-α*.* The data were analyzed by the one-way ANOVA, followed by Tukey’s *post hoc* test. (###) *p* < 0.001, significantly different when compared with that of the control group. (∗) *p* < 0.05, (∗∗) *p* < 0.01 and (∗∗∗) *p* < 0.001, significantly different when compared with that of the LPS group, ns: not significant. The data are representative of three independent experiments

To further confirm the above RT-qPCR data, we performed the ELISA analyses. The cells treated with FX alone showed no changes in TNF-α and IL-6 levels (**Figures 6A, B**), confirming that FX did not affect TNF-α and IL-6 levels. In the cells treated with LPS alone, the levels of TNF-α, IL-1β, and IL-6 increased significantly when compared with that of the sham group (*p <* 0.05). However, the FX+LPS co-treatment resulted in the suppression on the TNF-α and IL-6 levels (*p* < 0.01). Specially, FX treatment exhibited inhibition of IL-6 production in a dose-dependent manner with the 50% inhibition concentration (IC_50_) value of 2.19 ± 0.70 μM (**Table 1**).

**Table 1:** Anti-inflammatory effects of FX on LPS-stimulated RAW264.7 cells.

Compound	IC_50_ values (μM)^a^		
	IL-1β	IL-6	TNF-α
FX	91.36 ± 0.86	2.19 ± 0.70	>100

a IC50 values for FX are given in column IL-1β, IL-6, and TNF-α. Values >100 μM are considered to be inactive. The data are representative of three independent experiments.

### FX Treatment Inhibits NF-κB Pathway in LPS-Treated RAW264.7 Cells and LPS-Treated Mice

**Figure 7:**
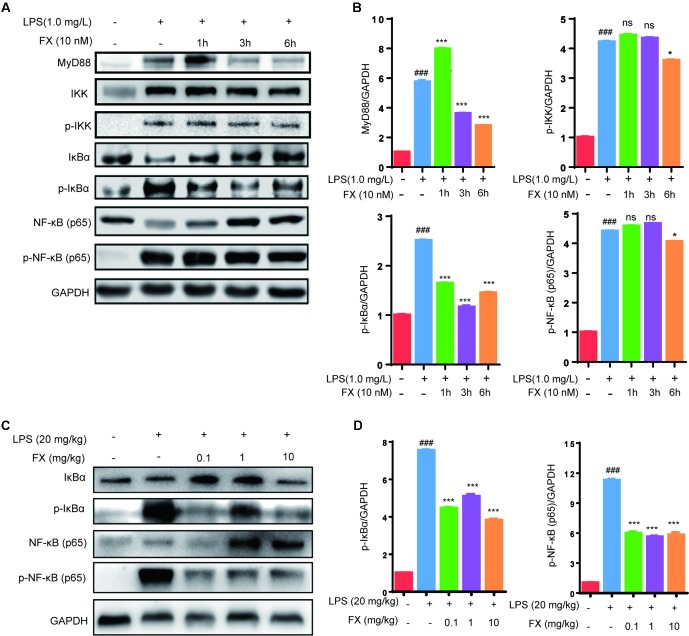
Effects of FX on NF-Κb signaling in LPS-treated RAW264.7 cells and LPS-treated mice. **(A)** Western blot analyses were performed to detect the levels of MyD88, IKK,p-IKK, IκBα, p-IκBα, NF-κB (p65), and p-NF-κB (p65) in LPS (1.0 mg/L)-treated RAW264.7 cells; **(C)** Protein levels of IκBα, p-IκBα, NF-κB (p65) and p-NF-κB (p65) in liver homogenates were evaluated by western blot analysis after LPS (20 mg/kg) challenge 6 h with or without FX treatment. **(B** and **D)** Densitometric analysis of the relevant bands was performed. The data were analyzed by the one-way ANOVA, followed by Tukey’s *post hoc* test. (###) *p* < 0.001, significantly different when compared with that of the control group. (∗) *p* < 0.05 and (∗∗∗) *p* < 0.001, significantly different when compared with that of the LPS group, ns: not significant. The data are representative of three independent experiments.

As NF-κB plays a central role in the regulation of inflammatory responses and macrophage functions, we evaluated its role in FX-mediated protective effects in RAW264.7 cells and in liver homogenates of LPS-treated mice. As shown in **Figures 7** (**A**, **B**), the RAW264.7 cells treated with LPS (1.0 mg/l) for 10 min resulted in a significant increase in the level of MyD88, IKK, p-IKK, p-IκBα and p-NF-κB (*p* < 0.05), indicating that LPS treatment activated the NF-κB-signaling pathway associated with inflammation. However, the co-treatment with FX (10.0 nM) for 6 h, followed by the administration of LPS (1.0 mg/L) at 10 min, resulted in significantly lower levels of MyD88, p-IKK, p-IκBα, and p-NF-κB when compared with those of the cells treated with LPS alone (*p* < 0.05). Similar findings also occurred in liver homogenates of LPS-treated mice. As shown in **Figures 7** (**C**, **D**), the inhibitory effect of FX on NF-κB pathway in LPS-treated mice is confirmed by showing that FX lowered the levels of p-IκBα and p-NF-κB in liver homogenates of LPS-treated mice.

**Figure 8:**
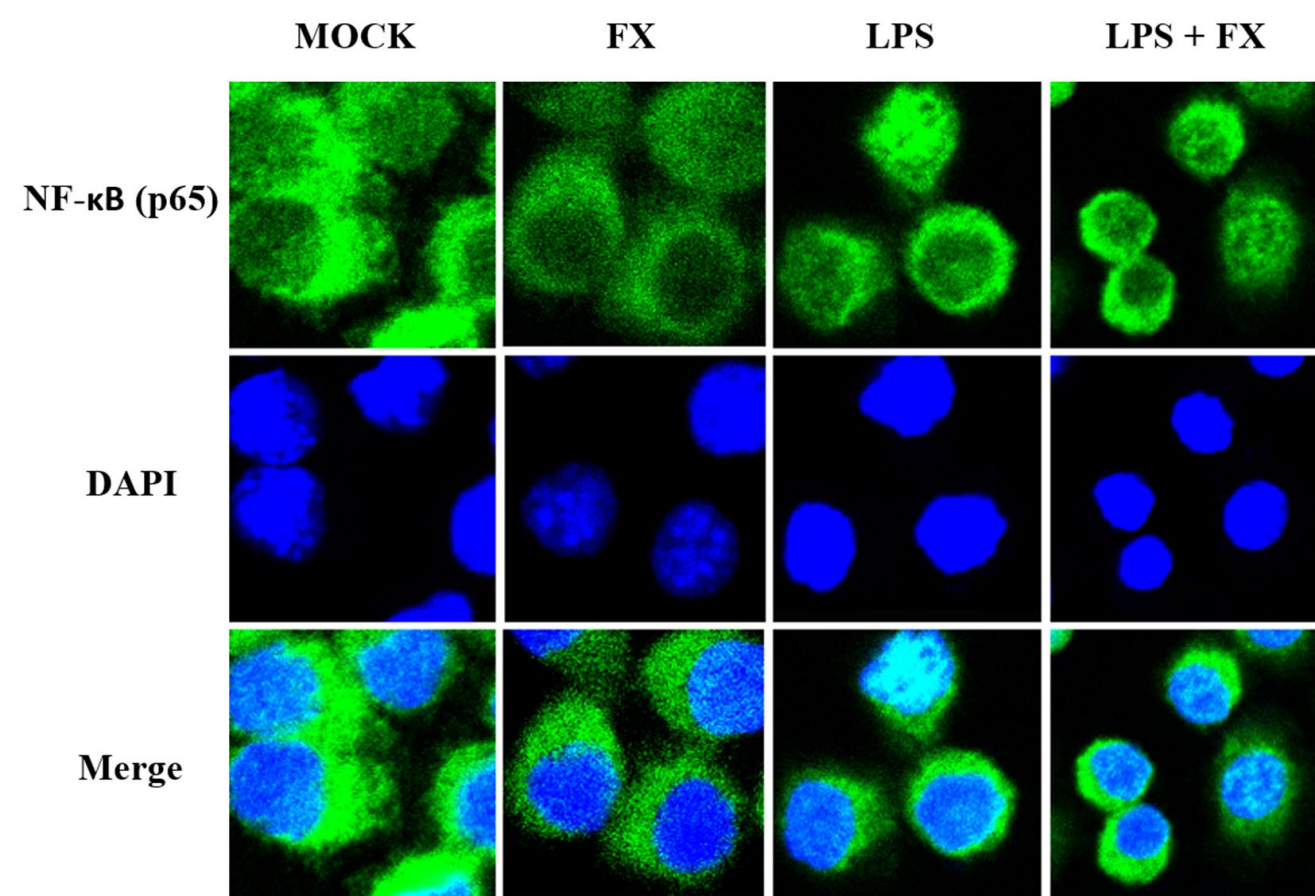
The effects of fucoxanthin (FX) on NF-κB nuclear translocation in RAW264.7 cell treated with LPS stimulus (scale bars: 20 μm). Immunofluorescence staining of NF-κB (p65) in RAW264.7 cell treated FX (10 nmol/L) at 3 h followed by LPS (1.0 mg/L) for stimulation. Immunofluorescence showed that FX inhibited NF-κB nuclear translocation upon LPS stimulation.

Since NF-κB will be translocated to the nucleus once it is activated, we next studied the effects of FX on the NF-κB translocalization by immunolabeling phospho-NF-κB (p65). As **Figure 8** showed, the immunomorphological findings indicated FX is able to inhibit NF-κB nuclear translocation upon LPS stimulation.

**Figure 9:**
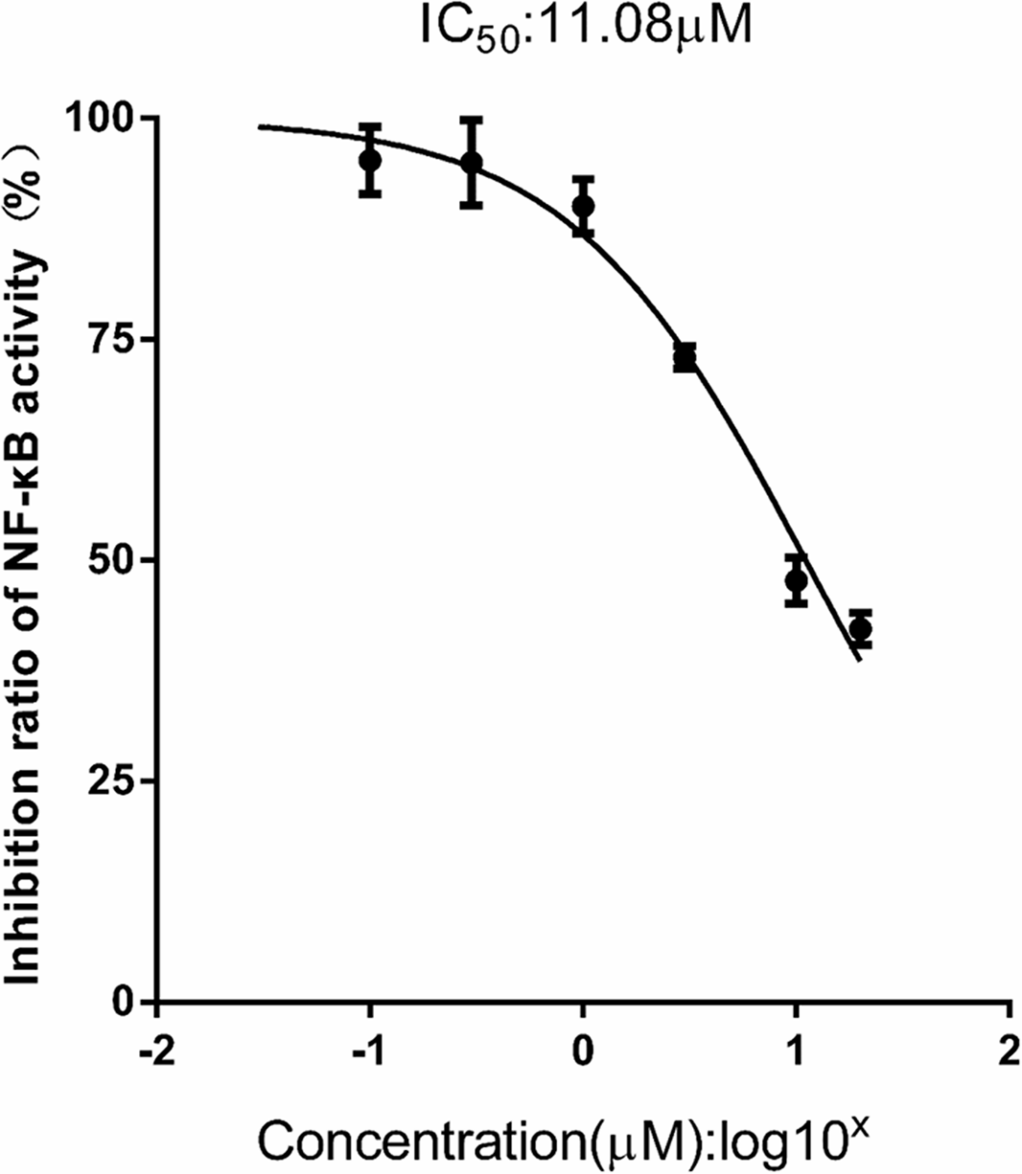
FX reduces NF-κB activity in THP1-Lucia™ NF-κB cells treated with LPS stimulus. THP1-Lucia™ NF-κB cells were pretreated with FX (0.03–30 μM) and then challenged with LPS (1.0 mg/L). FX showed a dose-dependent reduction in NF-κB activity in THP1-Lucia™ NF-κB cells stimulated with LPS (1.0 mg/L). Based on the logistic dose response curves, the 50% inhibitory concentration (IC_50_) of the FX in decreasing NF-κB activity was 11.08 ± 0.78 μM. n = 5 for each treatment group. The data are representative of three independent experiments.

Additionally, as shown in **Figure 9**, FX (0.03–30 μM) produced a significant dose-dependent reduction of NF-κB activity stimulated with LPS (1.0 mg/l), which indicated the IC_50_ inhibition of the expression of NF-κB was 11.08 ± 0.78 μM in the THP1-Lucia^TM^ NF-κB cells.

## Discussion

For the first time, the results of this study ultimately proved that the FX isolated from *C. weissflogii* ND-8 with the yield of 6 mg/gdw, and protected the mice against LPS-induced sepsis death with the survival rate from 0% to 40%, as well as FX showed dose-dependent inhibition against LPS-induced pro-inflammatory factor IL-6 production (IC_50_ = 2.19 ± 0.70 μM) *via* significantly does-dependently inhibited the LPS induced the upregulated expression of phosphor-IκBα and phosphor-NF-κB in RAW264.7 cells. Meanwhile, the dose–response curves indicated an estimated IC_50_ of 11.08 ± 0.78 μM FX for LPS stimulated THP1-Lucia^TM^ NF-κB cells.

Generally, the structures and contents of FX in large brown algae and microalgae differ. For instance, the content of FX is higher than carotene in brown algae (Zailanie and Purnomo, 2017). The seasonal growth character of large seaweeds (brown algae) has restricted the development and commercial utilization of FX. In the other hand, the microalgae grow rapidly and are abundant sources of FX. Particularly, the microalgae have the potential to produce high-value metabolites, and the production of their bioactive substances can be enhanced by manipulating their culture conditions. Thus, in recent years, microalgal culture has received increasing attention. The largest population of marine microalgae is diatoms, which are the key primary producers of marine ecosystems. They exhibit high biodiversity and enormous biomass, accounting for about 40% of the primary production in the oceans (Benavides et al., 2013; Buono et al., 2016). Among them, *C. weissflogii* is a representative species of marine diatoms and has a wide global distribution (Gardes et al., 2011). *C. weissflogii* has the characteristics of small size, short growth cycle, high survival rate, and anti-pollution ability. It is also an important economic food organism, which has a high nutritional value with high content of polyunsaturated fatty acids, such as DHA and EPA, thus exhibiting a positive effect on the growth and development of some shrimp seedlings (Kiatmetha et al., 2011).

Currently, the market price of FX is relatively high. FX contains multiple isomers, and different isomers have different physicochemical properties (Zhang et al., 2015). One of the main obstacles in the commercial production of FX is the low efficiency, selectivity, and high cost associated with the extraction and purification processes (Vieira et al., 2017). The commonly used purification methods include SGCC, HPLC, TLC, CPC, and HSCC (Kim et al., 2011; Xiao et al., 2012; Gomez-Loredo et al., 2015; Tokarek et al., 2016). In addition, the stability of FX is low. In the present study, we isolated a strain of ND-8 producing FX from the field and identified it as *C. weissflogii* by morphological and molecular biological approaches (**Figure 1**). FX was detected in the crude pigment extract of ND-8 by the HPLC-MS method, and the yield was 6 mg/gdw (**Figure 2**). FX-producing microalgae are distributed across the strains *Chaetoceros calcitrans, Chaetoceros gracilis, Cylindrotheca closterium, Isochrysis aff. Galbana, Isochrysis galbana, Phaeodactylum tricornutum, Nitzschia* sp.*,* and *Odontella aurita*. HPLC studies, including this one, have shown that the FX yield of microalgae ranges from 2.08 mg/g to 18.47 mg/g (Kim et al., 2012b; Xia et al., 2013; Foo et al., 2015; Gomez-Loredo et al., 2016; Wu et al., 2016; McClure et al., 2018). Therefore, we performed extensive culture, obtained FX samples from the crude extract of *C. weissflogii* ND-8 by silica gel column chromatography and TLC, and its high purity was determined by the NMR analyses (**Figure 3**) and biological activity was further confirmed. Thus, we have identified a new source that can be used in the production of FX.

According to epidemiological and clinical data, the bacteria, fungi, viruses, parasites, etc. are the main pathogens of sepsis. In the early stage of sepsis, with the help of lipopolysaccharide-binding protein (LBP), the LPS binds to the cluster of differentiation 14, an LPS receptor, to form an immune complex that ultimately interact with TLRs to promote intracellular signal transduction and release various inflammatory mediators such as IL-6, IL-1β, and TNF-α (Cohen, 2002; Guijarro-Munoz et al., 2014 Kumar, 2018; Lendak et al., 2018). As the research progresses, it found that LPS activates a wide range of signaling pathways, including JAK2, PI3K, STAT3, IRAK, and NF-κB signaling pathways. NF-κB nuclear translocation is key to immune-cell activation and proinflammatory cytokine expression, which LPS specifically activates the NF-κB transcription factors *via* TLR4 binding and activate the expression and release of inflammatory mediators such as IL-6, IL-1β, and TNF-α (Ntoufa et al., 2016).

We further studied the anti-inflammatory protective effect of FX isolated from the ND-8 strain using LPS-induced sepsis mouse and cell inflammation models. In the present study, we observed that FX can significantly inhibit the production of inflammatory cytokines IL-6, IL-1β, and TNF-α and promote the sepsis mouse model survival rate from 0% to 40% for the first time (**Figure 4**). The higher concentration (10.0 mg/kg) of FX worked better than the lower concentration (1.0 mg/kg) before 72 h treatments, probably consistent with the functional consequences due to the changes on the serum levels of inflammatory mediators. It has reported that no toxic effect was found for FX up to 2,000 mg/kg used (Beppu et al., 2009). However, there might be some toxic effects of FX at the higher concentration (such as 10.0 mg/kg) when combined with LPS due to some unknown metabolism. The question need to be further addressed in the future. We explored the function of FX on LPS-induced inflammatory response in mouse macrophage Raw264.7 cells. The results revealed that the FX-mediated anti-inflammation function is associated with the activation of NF-κB (**Figures 5**–**9**), which is consistent with the findings of previous studies, and have shown that FX can inhibit NO synthase and cyclooxygenase protein, and reduced the expression of NO and prostaglandin E2 levels, by inhibiting the activation of NF-κB and phosphorylation of MAPK (Kim et al., 2010). Numerous studies have reported that FX suppresses LPS-induced overexpression of iNOS and COX-2, as well as pro-inflammatory cytokines production *in vitro* and *in vivo* (Shiratori et al., 2005; Kim et al., 2010; Heo et al., 2012; Islam et al., 2013; Lee et al., 2013; Pangestuti et al., 2013; Robertson et al., 2015; Choi et al., 2016; Zhao et al., 2017; Jiang et al., 2018; Jung et al., 2018; Yang et al., 2018), which is similar to our findings. As the previous studies reported, the anti-inflammatory mechanism of FX involved NF-κB pathways (Kim et al., 2010b; Robertson et al., 2015; Zhao et al., 2017; Jiang et al., 2018). The results of our present study also proved that FX dose-dependently attenuates pro-inflammatory cytokine IL-6 expression in LPS-activated RAW264.7 microglia cells, and we first found that the IC_50_ was 11.08 ± 0.78 μM for the inhibition of the expression of NF-κB in the THP1-Lucia^TM^ NF-κB cells.

Previous studies have revealed that several small molecules, such as resatorvid (TAK-242), ibuprofen, aspirin, kukoamine b, donepezil, geniposide, corilagin, resveratrol, agmatine, leonurine, sinomenine, and crebanine, were all able to inhibit the production of inflammatory cytokines or increase the survival rate of septic model animals. Some studies have shown that their effects are dependent on NF-κB and STAT3 activation, while others have shown their effects on the PI3K signaling pathways (Rice et al., 2010; Eisen et al., 2012; Li et al., 2014; Shi et al., 2014; Xu et al., 2014; Heinbockel et al., 2015; Yi et al., 2015; Arikawa et al., 2016; Intayoung et al., 2016; Sui et al., 2016; Li et al., 2017). Therefore, the anti-inflammation functions might involve a variety of mechanisms. These results support the notion that FX is a promising agent for preventing sepsis by inhibiting the NF-κB pathways. Furthermore, the mechanism by which FX regulates the associated underlying molecular signaling pathways remain to be elucidated. Some specific inhibitors and CRISPR-Cas9 technology would be applied to prove the involvement of NF-κB pathways in the anti-inflammatory activity of FX in sepsis mouse modals and LPS-activated cells.

## Conclusions

In summary, the present study has established a method to extract FX from *C. weissflogii* ND-8 and explored the protective effect of FX in a prophylactic manner in a mouse model of sepsis and in Raw267.4 macrophage cells. FX exhibited protective effect in septic mice, by inhibiting the expression of inflammatory cytokines with the IC_50_ inhibition of IL-6 production, was 2.19 ± 0.70 μM in Raw267.4 macrophage cells. Our data reveal that the FX-induced anti-inflammatory functions are associated with the regulation of NF-κB signaling, with the IC_50_ inhibition of the expression of NF‐κB was 11.08 ± 0.78 μM in the THP1-Lucia^TM^ NF-κB cells. Thus, FX presents anti-inflammatory properties and has therapeutic potential toward treatment of sepsis.

## Data Availability

All datasets generated for this study are included in the manuscript and the supplementary files.

## Ethics Statement

Specific pathogen-free C57BL/6 adult mice aged 8–10 weeks old and weighing 20 ± 1 g were purchased from the Fujian Medical University Animal Facility (Fujian, China). All animal experiments were conducted in accordance to the Guide for the Care and Use of Laboratory Animals approved by the Fujian Provincial Office for Managing Laboratory Animals and was guided by the Fujian Normal University Animal Care and Use Committee (Approval No. 201800013).

## Author Contributions

JS, YC, LS, DL, KL-P, GW, LC, ZL, LH and QC conceived the experiments. JS and QC wrote the manuscript. JS, KG, YL, JZ and MH performed the main experiments. JS and KG statistically analyzed all data. All authors reviewed the manuscript.

## Funding

The present study was supported by the National Natural Science Foundation of China (Grant No. 81803547), the Natural Science Foundation of Fujian Province, China (Grant No. 2018J01720), Innovative Research Teams Program II of Fujian Normal University in China (Grant No. IRTL1703, FZSKG2018013), the Public Service Platform for Industrialization Development Technology of Marine Biological Medicine and Product of State Oceanic Administration (Grant No. FZHJ14), and Provincial University Project of Fujian Education Department (Grant No. JK2017020).

## Conflict of Interest Statement

The authors declare that the research was conducted in the absence of any commercial or financial relationships that could be construed as a potential conflict of interest.

## Abbreviations

COX-2, cyclooxygenase; ELISA, enzyme-linked immunosorbent assay; FX, Fucoxanthin; HPLC, high-performance liquid chromatography; HPLC-MS, high-performance liquid chromatography-mass spectrometry; iNOS, inducible NO synthase; IL, interleukin; ITS, internal transcribed spacer; LPS, lipopolysaccharide; MyD88, myeloid differentiation primary response gene 88; NF, nuclear factor; NMR, nuclear magnetic resonance; PCR, quantitative polymerase chain reaction; TLC, thin layer chromatography; TNF, tumor necrosis factor.
